# Cells at the Edge: The Dentin–Bone Interface in Zebrafish Teeth

**DOI:** 10.3389/fphys.2021.723210

**Published:** 2021-10-06

**Authors:** Joana T. Rosa, Paul Eckhard Witten, Ann Huysseune

**Affiliations:** ^1^Research Group Evolutionary Developmental Biology, Biology Department, Ghent University, Ghent, Belgium; ^2^Comparative, Adaptive and Functional Skeletal Biology (BIOSKEL), Centre of Marine Sciences (CCMAR), University of Algarve, Campus Gambelas, Faro, Portugal

**Keywords:** odontoblast, osteoblast, dentin, bone, tooth attachment, zebrafish, dermal skeleton, *scpp*

## Abstract

Bone-producing osteoblasts and dentin-producing odontoblasts are closely related cell types, a result from their shared evolutionary history in the ancient dermal skeleton. In mammals, the two cell types can be distinguished based on histological characters and the cells’ position in the pulp cavity or in the tripartite periodontal complex. Different from mammals, teleost fish feature a broad diversity in tooth attachment modes, ranging from fibrous attachment to firm ankylosis to the underlying bone. The connection between dentin and jaw bone is often mediated by a collar of mineralized tissue, a part of the dental unit that has been termed “bone of attachment”. Its nature (bone, dentin, or an intermediate tissue type) is still debated. Likewise, there is a debate about the nature of the cells secreting this tissue: osteoblasts, odontoblasts, or yet another (intermediate) type of scleroblast. Here, we use expression of the P/Q rich secretory calcium-binding phosphoprotein 5 (*scpp5*) to characterize the cells lining the so-called bone of attachment in the zebrafish dentition. *scpp5* is expressed in late cytodifferentiation stage odontoblasts but not in the cells depositing the “bone of attachment”. nor in *bona fide* osteoblasts lining the supporting pharyngeal jaw bone. Together with the presence of the osteoblast marker Zns-5, and the absence of covering epithelium, this links the cells depositing the “bone of attachment” to osteoblasts rather than to odontoblasts. The presence of dentinal tubule-like cell extensions and the near absence of osteocytes, nevertheless distinguishes the “bone of attachment” from true bone. These results suggest that the “bone of attachment” in zebrafish has characters intermediate between bone and dentin, and, as a tissue, is better termed “dentinous bone”. In other teleosts, the tissue may adopt different properties. The data furthermore support the view that these two tissues are part of a continuum of mineralized tissues. Expression of *scpp5* can be a valuable tool to investigate how differentiation pathways diverge between osteoblasts and odontoblasts in teleost models and help resolving the evolutionary history of tooth attachment structures in actinopterygians.

## Introduction

Bone and dentin were concomitantly present in the earliest elements of the dermal skeleton (Ørvig, 1951; Sire and Kawasaki, 2012; Keating et al., 2018). The first mineralized skeleton appeared in jawless vertebrates of the Ordovician, the pteraspidomorphs (“heterostracomorphs”, Donoghue and Sansom, 2002; Keating et al., 2018). Their body armor consisted of scales, ornamented with tubercles or ridges composed of a superficial layer of dentin, acellular bone, and, in some taxa, enameloid (Donoghue and Sansom, 2002). As a result of their shared evolutionary history, bone and dentin have many important characters in common (see e.g., Ørvig, 1951, 1967, and many references therein). Not surprisingly, the cells that secrete these matrices, osteoblasts and odontoblasts, are closely related cell types. This was already recognized by Klaatsch (1890, cited in Ørvig, 1951), who designated the term “scleroblast” for any cell participating in hard tissue formation.

In the mammalian jaw complex, osteoblasts and odontoblasts can usually easily be distinguished, as bone and dentin are well delimited anatomically and distinctive histologically. Mammalian teeth form discrete units with an internal cavity paved by odontoblasts. The root dentin that these cells produce is covered outward by cementum and delimited from the alveolar bone by the periodontal ligament. Within this tripartite periodontal complex, the tissues are kept well apart and are maintained through tightly balanced interactions (Fleischmannova et al., 2010). Different from mammals where tissue identification is commonly unambiguous, non-mammalian amniotes with ankylosed teeth possess an attachment tissue whose identification has raised considerable debate. It may either be related to cementum or to alveolar bone, but nevertheless appears to be clearly distinguishable from dentin (Bertin et al., 2018).

**Figure 1:**
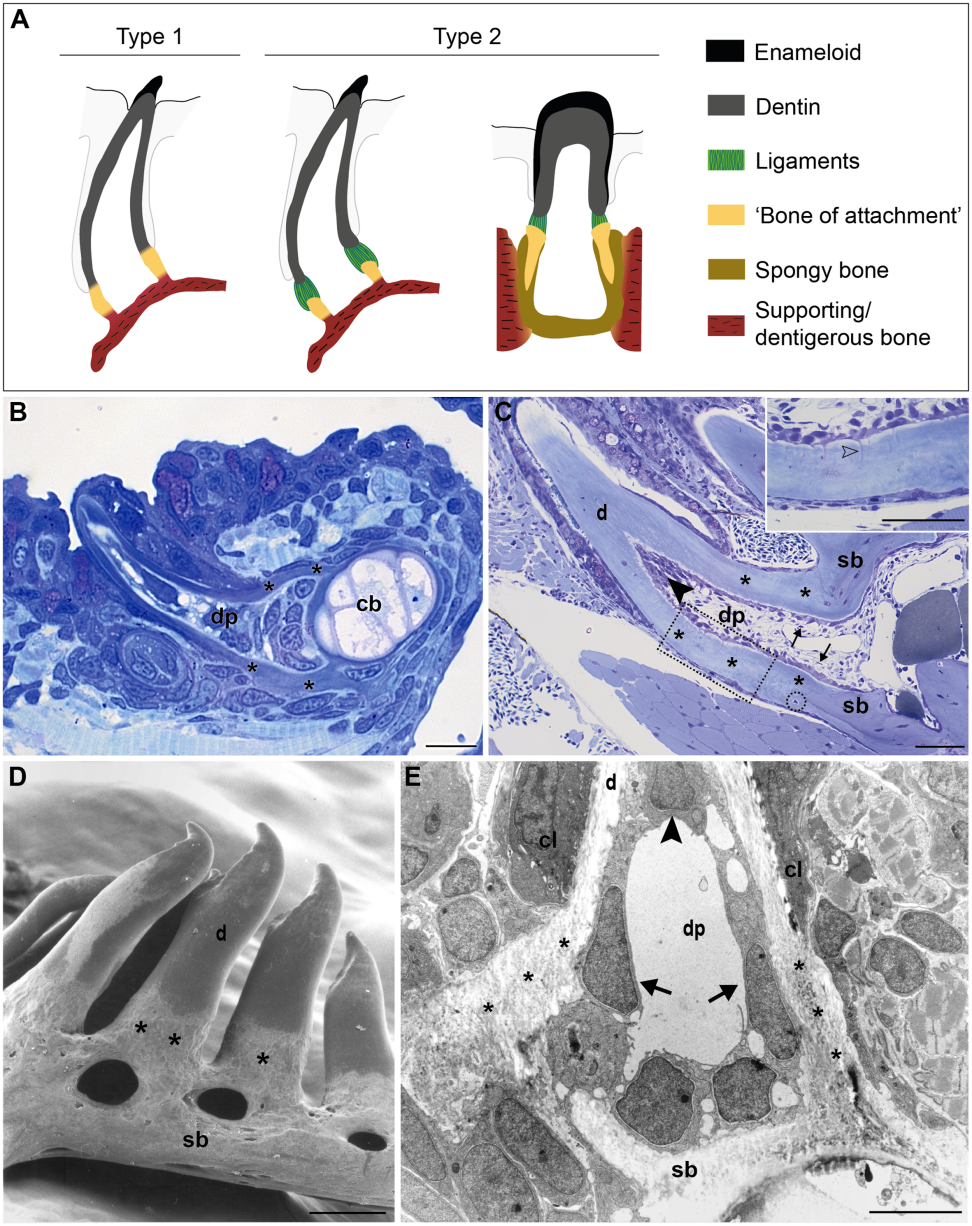
Tooth attachment in actinopterygians. **(A)** Schematic representation of type 1 and type 2 tooth attachment modes according to Fink (1981). In type 1 (e.g., zebrafish), a cylindrical collar of tissue, hitherto called “bone of attachment” (“attachment bone” in the terminology of Fink, 1981), firmly ankyloses the tooth to the supporting bone, resulting in continuous mineralization between the tooth base and the supporting (dentigerous) bone. In type 2, the cylindrical collar of “bone of attachment” is connected to the tooth by a ligament. The collar is positioned either on top, or inserted into the supporting bone. Note that in the latter case, Berkovitz and Shellis (2016) label the “bone of attachment” as “pedicel”, reserving the term “bone of attachment” for the tissue serving to attach the pedicels. Note that the term “bone of attachment” is used between quotation marks, pending a more appropriate term based on the findings in this study. **(B)** and **(C)** Toluidine blue-stained semi-thin sections of a zebrafish initiator tooth at 6dph **(B)** and an adult tooth **(C)**, both attached, as in type 1 attachment. Note multiple osteocytes in the adult supporting bone, a single one in the “bone of attachment” (encircled). Inset: enlargement of “bone of attachment” (dotted rectangle) with cell prolongations (open arrowhead). **(D)** Scanning electron micrograph of attached adult zebrafish teeth. **(E)** Overview transmission electron microscopy (TEM) picture of the base of a zebrafish first-generation tooth at the level of the cervical loop. The dentin, “bone of attachment” and supporting bone form a continuous mineralized tissue, covered along the pulpal side with scleroblasts that appear to be involved in the deposition of more than one matrix. cb, ceratobranchial cartilage; cl, cervical loop; d, dentin; dp, dental pulp; sb, supporting bone; asterisks, “bone of attachment”; arrows, scleroblasts forming “bone of attachment”; black arrowheads, odontoblasts. Scale bar **(B)**=10μm, **(C)**=50μm, **(D)**=100μm, and **(E)**=5μm.

The large group of teleost fish (about 30,000 species), on the other hand, features a broad diversity of tooth attachment modes, ranging from fibrous attachment of the dentin base to the underlying bone, to firm ankylosis (Gaengler, 2000). The part of the tooth that is not covered by the hypermineralized cap of enameloid is sometimes called a root (e.g., Bemis et al., 2005). However, the term “root” for teleost (or even actinopterygian) teeth is not widely accepted. Many teleosts have teeth anchored on top of the bone, either during the entire lifetime of the animal, or at least in the first tooth generations (Trapani, 2001; Sire et al., 2002). This type of attachment, or ankylosis, begins when the base of the elongating tooth germ approaches the jaw bone. Outgrowths of the tooth and the adjacent bone then form a composite tissue in which bone and dentin are in intimate contact (Berkovitz and Shellis, 2016). This is distinguished from gomphosis, or anchoring in an alveolus. Furthermore, unlike “rooted” teeth, teleost teeth are broadest in their basal area, generally with a wide open pulp cavity (Peyer, 1968). Therefore, the proximal part of teleost teeth is commonly labeled as “basal portion” or “base” (Peyer, 1968), or remains an unnamed part of the “shaft” (Berkovitz and Shellis, 2016). Here, we will adopt the term “base”. Fink (1981) distinguished four types of attachment in actinopterygians, with the participation of a histologically distinct structure, which he referred to as “attachment bone” (here referred to as “bone of attachment”, a term originally coined by Tomes, 1875, 1882). In his type 1 mode (Figure 1A), the mature tooth is completely ankylosed to the bone – that is, mineralization is continuous between the tooth base and the “bone of attachment”, a situation considered to be the primitive actinopterygian condition (Fink, 1981). For example, in zebrafish (a cyprinid, and a common developmental and genetic model species), the entire connection between the dentin base and the supporting, dentigerous, bone is mineralized (Figures 1B,C; Huysseune et al., 1998; Van der heyden et al., 2000). In contrast, many teleosts possess a small area of unmineralized collagen persisting as a ligament between the dentin base and the cylinder of hard tissue (“bone of attachment”; type 2 attachment in Fink’s classification, Figure 1A; Huysseune, 1983). In highly evolved teleosts with intramedullary tooth development, e.g., cichlids, this cylinder is deeply inserted into the jaw bone, and fused to the surrounding dentigerous bone *via* spongy bone (Figure 1A). Shellis (1982) and Berkovitz and Shellis (2016) reserve the term “bone of attachment” exclusively for type 1 attachment of Fink (1981). They call the cylinder of hard tissue, ligamentously connected to the dentin (Fink’s type 2 attachment), “pedicel”, and reserve the term “bone of attachment” for the bone tissue interconnecting pedicels or attaching pedicels to the jaw bone (Shellis, 1982; Berkovitz and Shellis, 2016; Figure 1A). The two other types described by Fink (1981) represent more specialized types of anchorage. Still other types of attachment exist (e.g., Soule, 1969; Bemis et al., 2005).

The nature of the tissue, attaching the tooth to the supporting bone in teleosts, whether the “attachment bone” of Fink (1981) or the “pedicel” of Shellis (1982) and Berkovitz and Shellis (2016), has been a matter of considerable discussion. In some species, it has been described as dentin (even if covered by osteoblasts on the outside). In such a case, the term “bone of attachment” would be inappropriate to describe this tissue (e.g., in sea bream species, Hughes et al., 1994). In other species, it has been claimed to have a bony nature, its development being initiated by the dentigerous bone (Clemen et al., 1997), justifying the term “bone of attachment”, or “attachment bone”. Perhaps the wide variety of dentins encountered in actinopterygians (Ørvig, 1967) may at least partly explain the structural diversity of the “bone of attachment”. Irrespective of the nature of the tissue, and whether partly or entirely mineralized, it presents as a cylinder (or collar) in the prolongation of the dentin base. Likewise, the layer of cells that secretes the dentin appears to extend from the pulp cavity along the inside of the “bone of attachment” down to the supporting bone (described for, e.g., zebrafish, Huysseune et al., 1998; sparids, Hughes et al., 1994; *Lophius*, Kerebel et al., 1979). As a result, it is unclear whether the cells that line the “bone of attachment” on its inside are odontoblasts, osteoblasts, or yet another (intermediate) type of hard tissue forming cell (“scleroblast”, Klaatsch, 1890, cited in Ørvig, 1951). Often, it is the assumed nature of the attachment tissue that is used to qualify the cells that produce this matrix as odontoblasts, or osteoblasts.

Given the close evolutionary, chemical and structural relationship of bone and dentin, it is not surprising that very few genes are expressed exclusively in just one of both cell types – osteoblasts or odontoblasts – rendering an unambiguous characterization of the attachment tissue difficult. Differentiation of epithelial cells into enamel/enameloid-producing cells, on the one hand, and mesenchymal cells into dentin-producing cells, on the other hand, relies on common genetic toolkits, namely genes encoding secreted signaling factors and their receptors, and transcription factors. Differences in their spatiotemporal pattern or strength of expression may alter the outcome. In contrast, extracellular matrix (ECM) proteins are more specific to mineralizing tissues and can thus be much more informative as to the structure of a particular tissue, hence the cell type that produces it (Kawasaki and Weiss, 2006). In 2009, Kawasaki (2009) reported the repertoire of secretory calcium binding phosphoproteins (SCPPs) in zebrafish and frogs and their expression in dental and skeletal tissues. The family of secretory calcium-binding phosphoproteins includes SCPPs involved in bone and dentin formation (the so-called acidic SCPPs or SIBLING proteins), as well as proteins involved in enamel formation, milk caseins and some salivary proteins (the so-called Pro/Gln (P/Q)-rich SCPPs; Kawasaki, 2009, 2011). Differential expression of SCPP genes in bone and dentin has been reported (see Kawasaki, 2009, 2011; Sire and Kawasaki, 2012 for an overview). Yet, only expression of *scpp5* was reported by Kawasaki (2009) to allow distinction between odontoblasts and osteoblasts in zebrafish. Thus, *scpp5* may be a good starting point to elucidate the nature of the “bone of attachment” in the zebrafish dentition.

In this paper, we examine the dentin-bone interface in the pharyngeal teeth and jaws of the zebrafish (the only tooth-bearing jaws in this species). We first highlight the resemblance between the different matrices, that is, dentin, supporting bone, and the collar of attachment tissue that has been termed “bone of attachment” in previous papers (Huysseune et al., 1998; Van der heyden et al., 2000). We next study the expression of *scpp5* in the different developmental stages of the initiator tooth (called tooth 4V^1^, Van der heyden and Huysseune, 2000; Gibert et al., 2019), other first-generation teeth, as well as adult teeth. Including both first-generation and adult teeth in the study is important because of substantial structural differences between the tooth generations. Indeed, first-generation teeth may well be more easily accessible for experimentation and (whole mount) gene expression studies, but they are extremely small, have an enamel organ and dental papilla containing a few cells only, and a pulp devoid of nerves and blood vessels. Unlike in their adult counterparts, their dentin is atubular, and supporting bone structures are still thin and virtually anosteocytic (Huysseune et al., 1998; Huysseune, 2000; Sire et al., 2002). The aims are (1) to reveal exactly where and in which stages of tooth development *scpp5* is expressed, both in early life stages and in the adult, and (2) to elucidate if the scleroblasts depositing the “bone of attachment” have an odontoblast or osteoblast character. These data are complemented by immunocytochemical data on the distribution of Zns-5, a cell surface antigen that has been used as an osteoblast-specific marker (Johnson and Weston, 1995). Together with structural data, the temporal and spatial distribution of *scpp5* and Zns-5 inform us on the nature of the cells producing the attachment tissue, and whether labeling it as “bone of attachment” is justified. This study contributes to the understanding of the character and the evolutionary history of tooth attachment in actinopterygians.

## Materials and Methods

### Zebrafish Maintenance and Breeding

Adult zebrafish (AB wild-type strain) were maintained and spawned according to Westerfield (2000). Embryos and early postembryonic stages were raised in egg water at 28.5°C and staged according to Kimmel et al. (1995). Early postembryonic stages from 72h post-fertilization (hpf) to 6 d post-fertilization (dpf), as well as 4-month-old adults were sacrificed by an overdose of 1% ethyl 3-aminobenzoate methanesulfonate (MS-222; E10521-10G, Sigma Aldrich) and further processed.

### Sample Processing for *in situ* Hybridization and Immunohistochemistry

Early postembryonic stages were fixed for 4h at 4°C in 4% paraformaldehyde [PFA; pH 7.4 in 1x phosphate-buffered saline in DEPC-H_2_O (PBS)], washed 3×5min with 1xPBS, dehydrated through a PBS/methanol gradient and stored in 100% methanol at 4°C. Prior to *in situ* hybridization, samples were rehydrated through an ascending methanol/PBS series. Pharyngeal jaws were dissected from adult zebrafish, fixed for 24h at 4°C in 4% PFA, rinsed in 1xPBS for 1h, and decalcified with 10% EDTA in Tris buffer-DEPC (100mmol, pH 7.2) at 4°C for 3weeks. Following dehydration, specimens were preserved at 4°C in 100% methanol until embedding. For paraffin inclusions, samples were first passed through an ascending methanol/xylene series, embedded in paraffin and then cross-sectioned (Microm HM360, Prosan). Sections (5μm thick) were collected on TESPA (3-aminopropyltriethoxysilane, Sigma-Aldrich) coated slides, dried for 4h at 37°C and kept at 4°C until further processed. For agar inclusions, samples were soaked in 5% sucrose in 1x PBS overnight and subsequently embedded in 1.5% agar/5% sucrose in 1x PBS. After solidifying, the blocks were transferred to 30% sucrose in PBS and kept at 4°C overnight. Blocks were then cross-sectioned on a cryotome (11μm thick; Shandon cryotome FSE), the sections collected on TESPA-coated slides, allowed to dry, and stored at −20°C until use.

For *in situ* hybridization, sense and antisense RNA probes were generated from 1μg of linearized pCR2.1-TOPO plasmid containing *scpp5* complete cDNA (EU642611) using T7 or SP6 polymerases, and then labeled with digoxigenin-dUTP (DIG RNA labeling kit, Roche Diagnostics, Mannheim, Germany). Riboprobes were treated with RNase-free DNase, recovered by ethanol precipitation and their integrity assessed through agarose gel electrophoresis. Whole mount *in situ* hybridization (used for early stages) was performed following the protocol described in Verstraeten et al. (2012). These specimens were then dehydrated in an ascending series of PBS/ethanol, embedded in epon and serially sectioned at 2μm. *In situ* hybridization on paraffin sections was performed using a protocol previously described by Rosa et al. (2016).

Immunohistochemistry on cryosections was performed using a monoclonal mouse anti-Zns-5 primary antibody (1:100, AB_10013796, ZIRC) and an Alexa Fluor® 594 (goat anti-mouse IgG, 1:200, Abcam) as secondary antibody. Negative controls were performed by omitting the primary antibody from the reaction mixture. Briefly, sections were thawed for 30min at room temperature, washed 3×20min in 1x PBS, permeabilized with acetone for 7min at −20°C and washed 2×15min in pre-blocking solution (0.5x PBS with 1% BSA, 1% DMSO and 0.5% Triton). Sections were then blocked for 1h 30min in blocking solution (pre-blocking solution with 1.5% goat serum) and incubated overnight at 4°C with the primary antibody diluted in blocking solution. The day after, sections were washed 8×10min with pre-blocking solution and incubated with secondary antibody overnight at 4°C. Finally, sections were washed with PBT (1x PBS with 0.3% Triton) and mounted with DAKO mounting medium (Agilent, Ref. S3023).

### Histology, Scanning and Transmission Electron Microscopy

Specimens for scanning electron microscopy (SEM) were prepared from cleared and stained adult jaws [maceration: 1–2% KOH; staining: 0.1% alizarin red S (Sigma) in 0.5% KOH]. After dehydration through a graded series of ethanol, specimens were dried (critical point drying, Balzers, CPD 020) and gold-coated (Balzers, SCD 040). Specimens were observed under a Jeol JSM-840 scanning electron microscope, operating at 15kV. For high resolution histology and transmission electron microscopy (TEM), samples were fixed in a mixture of 1.5% PFA and 1.5% glutaraldehyde as previously described (Huysseune and Sire, 1992), embedded in epon, serially sectioned at 1μm, and stained with toluidine blue. Ultrathin sections were prepared on an ultratome (Reichert Ultracut E), contrasted with uranyl acetate and lead citrate, and observed in a Philips 201 transmission electron microscope operating at 80kV. Tissues and cellular structures in sections of adult specimens were identified through Heidenhain’s Azan-staining following the protocol described in Romeis (1989). Sections were observed on a Zeiss Axio Imager Z1^1^. Photomicrographs were taken with an MRC camera and processed using ZEN software (Zeiss; see foot note 1). Computer-generated images were processed for color balance, contrast, and brightness only, and applied to all parts of the figures equally.

### Ethical Statement

Animal care and sacrifice complied with European Directive 2010/63/EU of 22 September, 2010. The experimental protocols involved euthanasia only (no animal experiments); all animal procedures used in this study were approved by Flemish authorities (laboratory permit number LA1400452).

### Data Availability Statement

All sections used for this study are kept in the slide collection of the Research Group “Evolutionary Developmental Biology” at the Biology Department of Ghent University and are available for inspection upon request.

## Results

### Dentin, “Bone of Attachment”, and Supporting Bone

In zebrafish, a collar of mineralized tissue, hitherto called “bone of attachment”, ankyloses the mature tooth to the supporting, dentigerous bone of the fifth ceratobranchial (type 1 attachment, Figure 1A), both in first-generation teeth (Figure 1B) as well as in later tooth generations and adult teeth (Figure 1C). Note that the term “bone of attachment” will be used here between quotation marks, pending the identification of the cells producing this tissue. The prospective site of formation of the “bone of attachment” becomes visible once the dentin cone has obtained its full length. A collagenous matrix is then deposited in the prolongation of the tooth base, from the level of the cervical loop down to the surface of the supporting bone (Huysseune et al., 1998; Van der heyden et al., 2000). Mineralization appears to occur instantaneously and fast throughout the “bone of attachment”. Once mineralized, the “bone of attachment” forms a continuous structure, connecting the base of the dentin to the dentigerous bone, and is almost indistinguishable from either of these matrices (Figures 1B,C). A scanning electron micrograph of attached adult teeth (Figure 1D) confirms the continuous connection between the dentin, the “bone of attachment” and the underlying supporting bone of the fifth ceratobranchial. However, the “bone of attachment” distinguishes itself by its pitted surface from the smooth surface of the dentin. In this character, the “bone of attachment” resembles the dentigerous bone matrix rather than the dentin, as can also be appreciated from a low magnification TEM micrograph (Figure 1E). At an ultrastructural level, dentin and “bone of attachment” can be distinguished based on the organization of the collagenous matrix, which coincides with the position of the cervical loop tip (Figure 1E, and see Huysseune et al., 1998; Van der heyden et al., 2000 for more details). In the dentin, the collagen fibrils are homogeneously distributed and preferentially oriented along the long axis of the tooth. In contrast, the “bone of attachment”, as well as the dentigerous bone, presents a woven-fibred matrix in which patches of electron-dense ground substance can be observed (Figure 1E). Yet, while the dentigerous bone contains osteocytes, at least when it has reached a sufficient thickness, the “bone of attachment” is virtually free of osteocytes, except for an occasional cell close to where the “bone of attachment” joins the dentigerous bone (Figure 1C). Most remarkably, however, the cells that line the three matrices inside the dental pulp do not appear to respect strict demarcations. In large (adult) teeth, the cells lining the dentin and the “bone of attachment” form an uninterrupted layer and the cells facing the “bone of attachment” present small prolongations not unlike dentinal tubules (Figure 1C, inset). These tubules, likely one per cell, extend perpendicular for some distance through the dentin and are unbranched. Their length decreases in a proximal direction. In first-generation teeth, tubules are absent, both in the dentin and the “bone of attachment”. Interestingly, a single cell can be found to be positioned adjacent to both dentin and “bone of attachment” (Figure 1E). Below, another cell, with different shape, adjoins both the “bone of attachment” and the dentigerous bone. Clearly, neither position nor morphology of these cells allows to establish their identity, let alone infer their role in the formation of dentin or the “bone of attachment”. Similar difficulties for establishing boundaries at the tooth base have been encountered in medaka (Larionova et al., 2021).

### *scpp5* Expression in First-Generation Teeth and Adult Teeth

**Figure 2:**
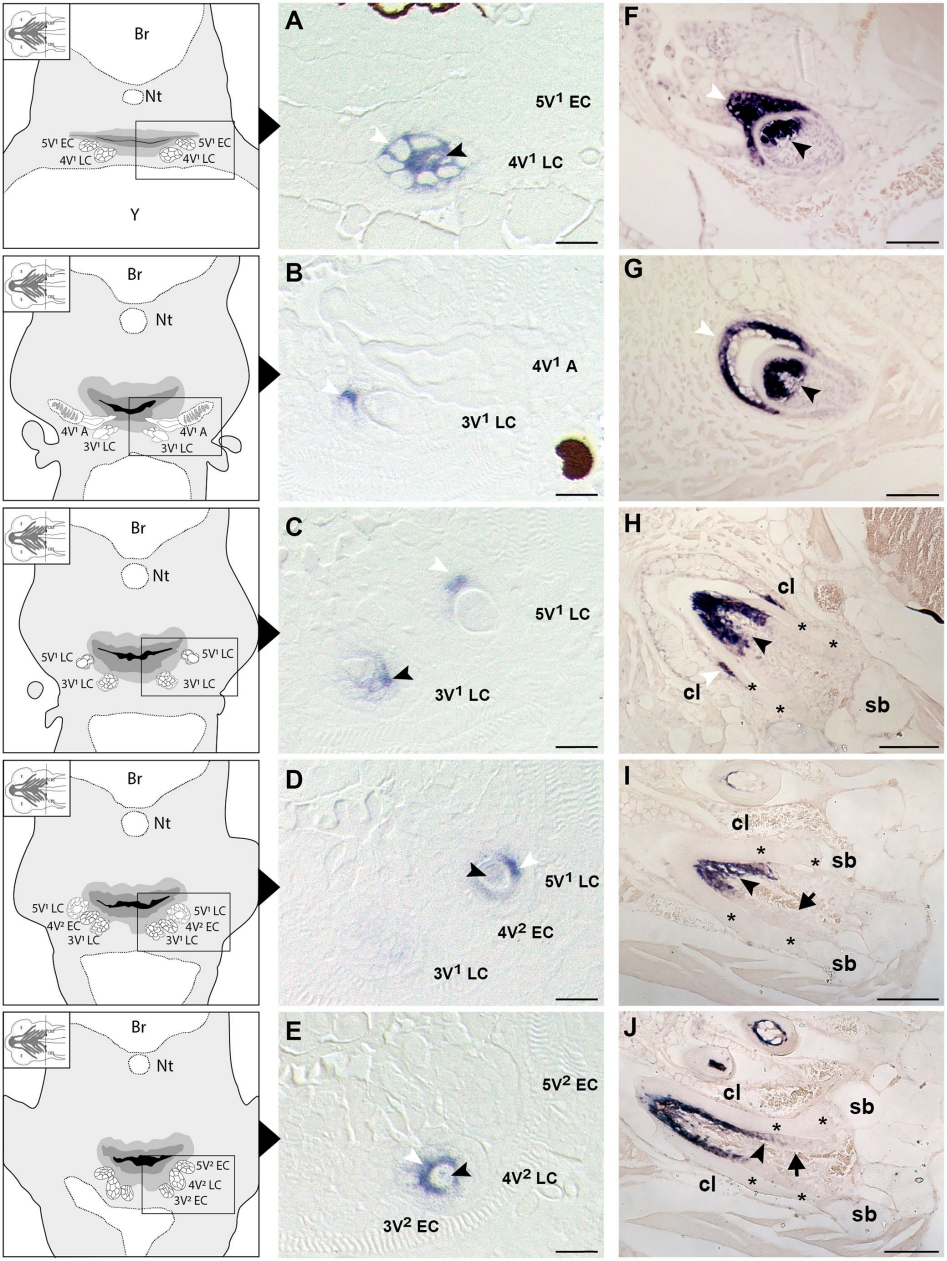
Expression of secretory calcium-binding phosphoprotein 5 (*scpp5*) during zebrafish tooth development. Transverse sections of zebrafish postembryonic stages (72–120hpf – **A–E**) and adults **(F–J)** in the region of the pharyngeal jaws, with explanatory schemes for postembryonic stages. At 72hpf **(A)** the initiator tooth (4V^1^) at LC stage expresses *scpp5* in both ameloblasts and odontoblasts, whereas no expression is detected for the first-generation teeth 3V^1^ and 5V^1^, still at EC stage. Between 80 **(B)** and 96hpf **(C)** tooth 4V^1^, now attached, shows no expression of *scpp5*, while 3V^1^ and 5V^1^ at LC stages start to express *scpp5* in both ameloblasts and odontoblasts. Between 96 **(D)** and 120hpf **(E)** the second-generation teeth (4V^2^, 3V^2^, and 5V^2^) fail to express *scpp5* during EC but upregulate expression in both ameloblasts and odontoblasts at LC. During the development of adult teeth, the expression of *scpp5* is detected in both ameloblasts and odontoblasts at EC **(F)**, LC **(G)** and when the tooth starts to attach to bone **(H)**. Expression is restricted to odontoblasts when the tooth is completely attached [**(I)** and **(J)**]. Tooth developmental stages: A, phase of attachment; EC, early cytodifferentiation; LC, late cytodifferentiation. Br, brain; cl, cervical loop; Nt, notochord; sb, supporting bone; Y, yolk; asterisks, “bone of attachment”; arrows, cells forming “bone of attachment”; white arrowheads, ameloblasts; black arrowheads, odontoblasts. Scale bar **(A-E)**=10μm, **(F–G)**=50μm, **(H–J)**=100μm.

To learn whether the cells depositing the “bone of attachment” are odontoblasts, osteoblasts, or yet another (intermediate) type of hard tissue forming cell, we have turned to *scpp5* as a specific marker of odontoblasts (Kawasaki, 2009). Zebrafish teeth go through five successive developmental stages: initiation, morphogenesis, early and late cytodifferentiation, and attachment (Huysseune et al., 1998; Van der heyden et al., 2000; Borday-Birraux et al., 2006). *scpp5* transcripts are first detected at the late cytodifferentiation (LC) stage of the initiator tooth (4V^1^), around 72hpf, in both ameloblasts and odontoblasts (Figure 2A). However, once 4V^1^ is attached, between 80 and 96hpf, the expression of *scpp5* is no longer detected in any of the cells of the dental unit, neither in the structures adjoining the tooth (Figure 2B). This profile of expression is also observed for the first-generation teeth adjacent to 4V^1^ (i.e., 3V^1^ and 5V^1^). A strong and specific signal from the *scpp5* riboprobe is observed in ameloblasts and odontoblasts at the LC stage of 3V^1^ and 5V^1^ (Figures 2B–D), but no signal is detected at early cytodifferentiation (EC; Figure 2A) or attachment (data not shown) stages, nor in any other cell types. Between 96 and 120hpf the second generation of teeth starts to develop. Again, *scpp5* transcripts are only detectable at LC stages and restricted to ameloblasts and odontoblasts (Figures 2D,E). In adult teeth, the expression of *scpp5* has a slightly different temporal pattern. Already during the stage of morphogenesis, both ameloblasts and odontoblasts express *scpp5*, an expression that is maintained during EC (Figure 2F) and LC (Figure 2G). When the teeth start to attach (Figure 2H), odontoblasts maintain *scpp5* expression but the expression by the ameloblasts is downregulated and it is only detectable at the tip of the cervical loop. Once the tooth is fully attached, ameloblasts completely cease to express the gene and its expression becomes restricted to the odontoblasts (Figures 2I,J). Most importantly, at no moment is expression of *scpp5* detected in osteoblasts adjacent to the dentigerous bone or in cells lining the “bone of attachment”. Instead, there is a well-defined separation between *scpp5*-expressing odontoblasts in the pulp cavity, down to where the cervical loop marks the boundary of the dentin, and the adjacent cells, that line the “bone of attachment” internally, and that do not express the gene.

### Zns-5 Expression in Adult Teeth

**Figure 3:**
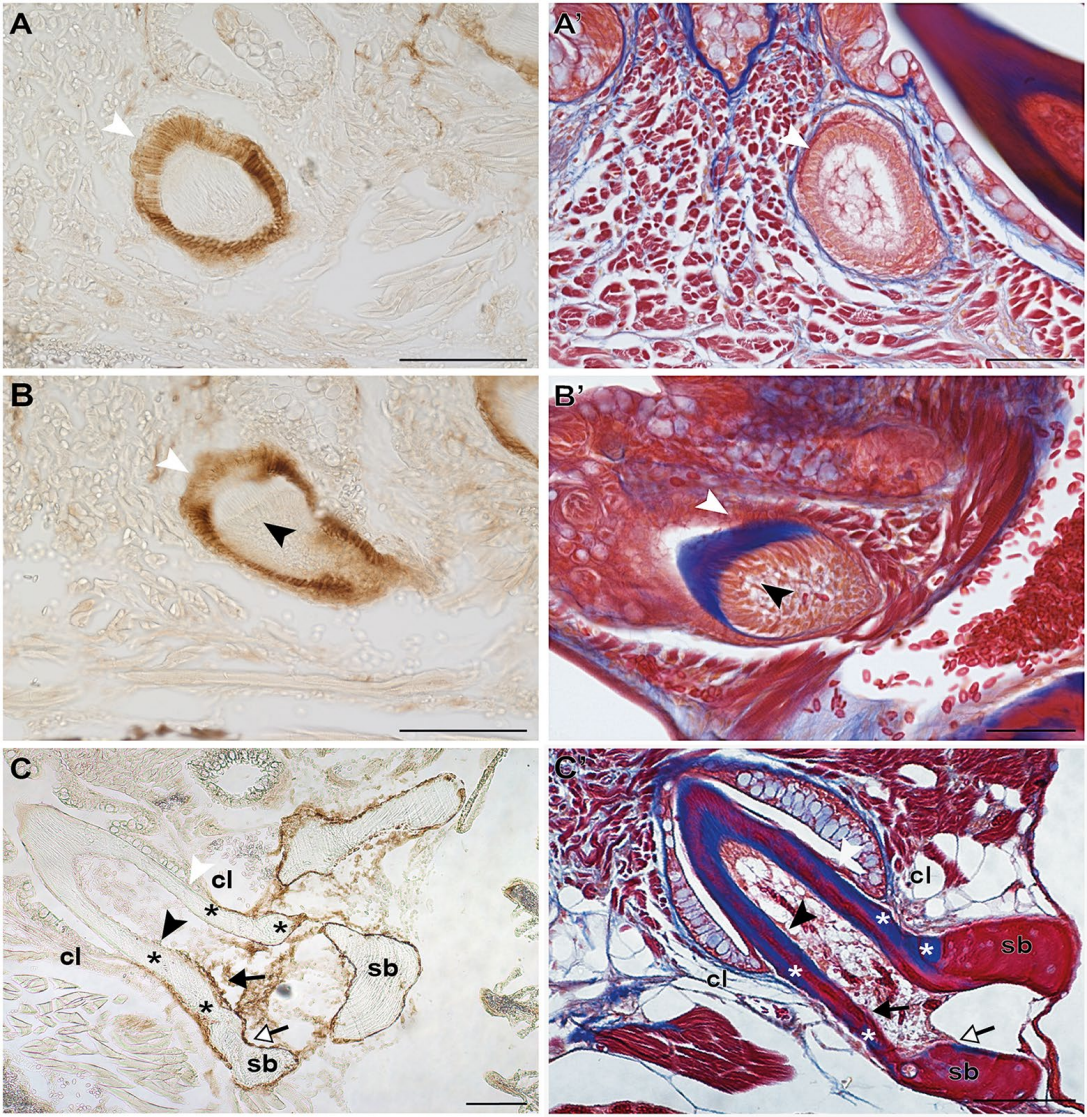
Zns-5 immunodetection during adult zebrafish tooth development. Transverse sections of adult zebrafish in the region of the pharyngeal jaws, used for Zns-5 immunohistochemistry **(A–C)** or stained with azan **(A’–C’)**. During EC **(A,A’)** and LC **(B,B’)**, Zns-5 is localized in ameloblasts, with no signal detected in odontoblasts. When teeth are attached **(C,C’)** ameloblasts cease to stain for Zns-5 and the antigen is only detected in the cells lining the “bone of attachment” and the osteoblasts lining the supporting bone. Note osteocytes in the supporting bone, but their absence in the “bone of attachment” **(C’)**. cl, cervical loop; sb, supporting bone; asterisks, “bone of attachment”; black arrowheads, odontoblasts; black arrows, cells forming “bone of attachment”; white arrowheads, ameloblasts; white arrows, osteoblasts. Scale bar **(A–D, C,’D’)**=100μm, **(A’,B’)**=50μm.

Irrespective of their differentiation status, osteoblasts in zebrafish can be specifically labeled by immunohistochemistry for the cell surface antigen Zns-5 (Johnson and Weston, 1995; Knopf et al., 2011). To elucidate if the cells that line the “bone of attachment”, and that do not express *scpp5*, have an osteogenic character, Zns-5 immunostaining was applied to adult zebrafish teeth of different developmental stages (Figure 3). The osteoblasts lining the supporting, dentigerous bone are specifically and strongly labeled both in areas where bone resorption already occurred and near to newly formed dentigerous bone (Figures 3C,C′). Importantly, the cells lining the “bone of attachment”, both on its internal and external surface, are also labeled by Zns-5 (Figures 3C,C′). These cells form an uninterrupted layer that connects with the labeled osteoblasts lining the supporting bone. Importantly, Zns-5 positive cells were never found distal to the level of the cervical loop. Instead, a clear boundary exists between the cells lining the “bone of attachment”, that are positive for Zns-5, and the adjoining odontoblasts, that face the dentin and are not labeled by the Zns-5 antibody. Interestingly, and despite the previously reported specificity of Zns-5 for osteogenic cells, Zns-5 was also found to label ameloblasts at I and M, EC, and LC tooth developmental stages (Figures 3A,A′,B,B′).

## Discussion

Our results clearly show that in the zebrafish pharyngeal jaw complex, odontoblasts are *scpp5* positive and Zns-5 negative, but cells lining the “bone of attachment”, like the osteoblasts, are *scpp5* negative and Zns-5 positive. However, unlike bone, the “bone of attachment” is predominantly anosteocytic, and the matrix contains small cell prolongations not unlike dentinal tubules, making it resemble dentin. These results suggest that the “bone of attachment” in zebrafish has characters that make an unambiguous assignment to either dentin or bone very difficult. We suggest the term “dentinous bone” to reflect the ambiguous nature of the tissue. The term “pedicel” may then replace “bone of attachment” when referring to the anatomical entity that it represents. In this way, we follow the terminology for type 2 as employed by Berkovitz and Shellis (2016), but propose to use the term “pedicel” instead of “bone of attachment” also for the attachment structure in type 1, based on assumptions of homology (unpublished results). The term “pedicel” does not imply a qualification of tissue type. It is dentinous bone in zebrafish, but may well qualify as dentin in other teleosts (e.g., in sea bream species, Hughes et al., 1994).

Various studies on actinopterygian teeth have shown that the deposition of tooth matrix continues uninterruptedly from the base of the dentin cone toward the supporting bone (e.g., Kerr, 1960; Huysseune et al., 1998; Van der heyden et al., 2000; Huysseune and Witten, 2008). Thus, one could have raised the question, at the onset of the study, whether the dentin cone does not simply extend toward the bone surface, and subsequently fuses to the bone, making the cylinder of attachment tissue simply part of the dental unit. For example, the description of tooth attachment in salmon (and for that matter in other basal actinopterygians) by Moy-Thomas (1934) specifies that there is no intermediate structure and that teeth are attached to the bone directly or to upgrowths more or less continuous with the bones. The cylinder of attachment tissue has indeed been assigned to the dental unit by various authors. Huysseune (1983) considered the collar of “bone of attachment” in a cichlid (a highly evolved teleost) as part of the dental unit. Likewise, Smith and Hall (1993) included the “bone of attachment” within the fundamental odontogenetic unit. Butler (1995) also considered “bone of attachment” to be derived from the mesenchyme of the dental papilla. However, being part of the dental unit does not automatically mean that the cylinder of attachment tissue is also dentin. In fact, the question whether there is truly a separate entity that merits a study, must be answered positively.

**Figure 4:**
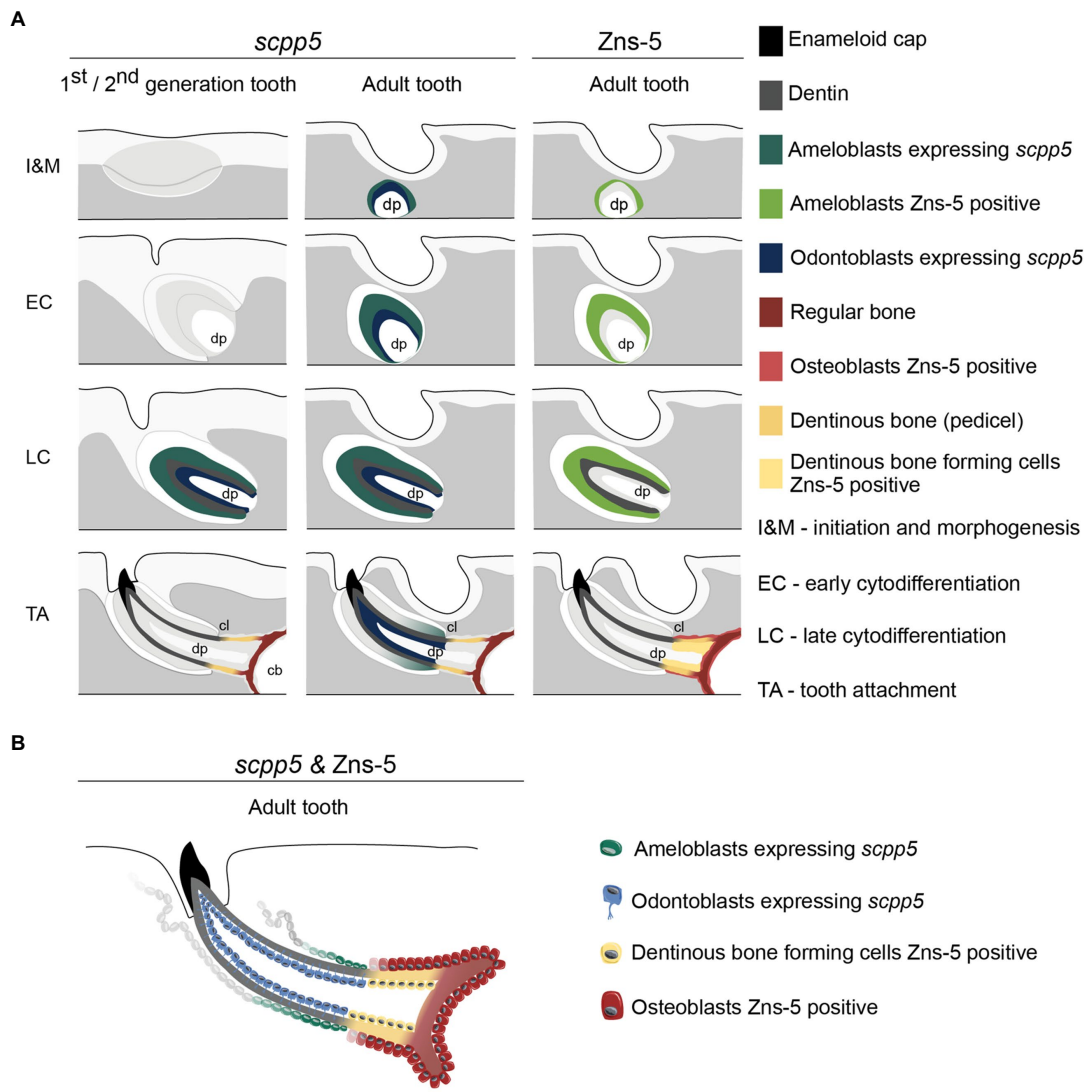
Interpretative scheme showing localization of *scpp5* transcripts and Zns-5 detection during zebrafish tooth development. Panel **(A)** shows a representation of *scpp5* expression during first and second generation and adult tooth formation, from the stage of initiation and morphogenesis to tooth attachment, and of Zns-5 detection in adult teeth, at the same developmental stages. Panel **(B)** shows the comparison between the expression of *scpp5* and the localization of Zns-5, the former being exclusively expressed by ameloblasts and odontoblasts and the latter by cells forming the pedicel and by osteoblasts. In both panels, the adult tooth is depicted in a similar way as the first/second-generation teeth, although the tooth is larger, with thicker walls, and is attached to bone with all cartilage having been resorbed in this area. cb, ceratobranchial cartilage; cl, cervical loop; and dp, dental pulp.

The data presented here, along with literature reports, provide several arguments in favor of the idea that a structure that is *not* true dentin connects the tooth to the supporting bone (summarized in Figure 4). First, the cervical loop delimits the extent of the epithelium and thus the direct interactions that can take place with odontoblasts *via* the basal lamina. One can argue that dentin is only produced where mesenchymal cells are covered by epithelium (Sire and Huysseune, 2003). That dentin development – in contrast to bone tissue – requires the proximity of an epithelium was also recognized by Ørvig (1967) and more recently by Sire and Huysseune (2003) and Sire and Kawasaki (2012). Both Osborn (1984) and Berkovitz and Shellis (2016) accepted the significance of the epithelium but reached a somewhat different conclusion. Osborn (1984), referring to description of tooth attachment of Shellis (1982) in the ballan wrasse, *Labrus bergylta*, recognized that odontoblasts differentiate under the influence of the epithelium (termed Hertwig’s root sheath, HRS, both by Shellis, 1982, and Osborn, 1984), but at the same time argued that the cylinder of root dentin is continued beyond HRS by cells differentiated from the base of the dental papilla. He called this tissue “attachment dentine”, and distinguished this from the bone-like tissue (“bone of attachment”), surrounding the base and produced by cells derived from soft tissue surrounding the developing tooth. Berkovitz and Shellis (2016) highlighted the ongoing discussion about the role (and potential retraction) of the epithelial sheath in the development of the attachment structures. They concluded that, whatever the role of the epithelial sheath, the pedicel is a joint product of the internal odontoblasts and the osteoblasts which cover the outer surface. Similar discussions have been ongoing regarding the role of Hertwig’s root sheath and the origin and nature of cementoblasts depositing acellular or cellular cementum during root formation in mammals (e.g., Bosshardt, 2005; Yamamoto et al., 2016).

Second, we now provide evidence that the cells that deposit the pedicel differ in their expression profile from *bona fide* odontoblasts. To start, we show that *scpp5* is exclusively expressed in developing teeth, confirming the observations of Kawasaki (2009) that the gene is specific for odontogenic, but not osteogenic cells. Likewise, based on RNA-seq data collected from the cichlid *Astatoreochromis alluaudi*, Karagic et al. (2020) found that *scpp5* is upregulated on tooth-bearing in contrast to nontoothed gill arches. Kawasaki (2009) showed the gene to be expressed both in the inner dental epithelium (IDE)/ameloblasts and odontoblasts but did not report anything on the pedicel. Here, we confirm the expression of *scpp5* in the ameloblasts and odontoblasts (at least in late cytodifferentiation stage). At no time did we observe any transcripts in the osteoblasts lining the dentigerous bone, or, for that matter, any other bone in the cranial and postcranial skeleton (data not shown). Most importantly, like true osteoblasts, the cells lining the pedicel do not express the gene. In this sense, these scleroblasts resemble osteoblasts. Moreover, there is a clearcut boundary between the proximalmost odontoblast, that expresses *scpp5*, and the distalmost scleroblast, that does not express the gene. Together with the presence of the osteoblast-specific marker Zns-5 in these scleroblasts, and its absence in odontoblasts, this strongly indicates that the cells lining the pedicel resemble osteoblasts rather than odontoblasts. A more detailed characterization of these cells would benefit from expression studies of other markers, such as *runx2*, a strong transcriptional activator for osteoblast-specific genes, and *sp7*, a transcription factor directly regulated by *runx2* (Komori, 2019). Both have been characterized as markers for zebrafish osteoblasts (e.g., Li et al., 2009; DeLaurier et al., 2010). Of relevance, *runx2* is expressed also in zebrafish odontoblasts (Kague et al., 2012, 2018) and *sp7* in zebrafish odontoblasts, osteoblasts and cells lining the “bone of attachment” (Kague et al., 2018). The studies of Kague et al. (2012, 2018) nevertheless rely on promotor-driven transgene expression; papers detailing endogenous *runx2* or *sp7* expression in odontoblasts of zebrafish (or any other teleost species) are currently lacking. However, various studies have shown that there is a close correspondence between endogenous expression of *sp7* (*via in situ* hybridization or antibody staining) and promotor-driven transgene expression (Renn and Winkler, 2009; DeLaurier et al., 2010; de Vrieze et al., 2015; Ando et al., 2017). A similar matching between endogenous and transgene expression has been observed for *runx2b* (Knopf et al., 2011). It is not excluded that a detailed analysis of endogenous *runx2* or *sp7* expression might reveal more specific expression patterns, restricted to certain mesenchymal cell types in the teeth. However, *sp7* is also expressed in zebrafish chondrocytes (Hammond and Schulte-Merker, 2009), as are the zebrafish *runx2* orthologs (Flores et al., 2006). The emerging picture of a chondrocyte-to-osteoblast lineage continuum (Beresford, 1981; Roach et al., 1995; Yang et al., 2014), along with the bone-dentin-enamel(oid)-continuum (Kawasaki and Weiss, 2008; Kawasaki, 2009), and the insight that bone- and dentin-forming cells are part of a unique cell population (Sire and Kawasaki, 2012), makes it unlikely that cells intermediate between odontoblasts and osteoblasts will differentially express these transcription factors. Fine scale studies required to uncover possible subtle differences are furthermore rendered difficult by the small cell size and number of odontoblasts in teleost fish, in some cases down to as little as one odontoblast (Larionova et al., 2021).

Unlike the supporting bone, on the other hand, the pedicel is largely devoid of osteocytes. If, as argued above, the pedicel is deposited by cells that resemble osteoblasts, suggesting the matrix deposited could be more bone-like, then there is a need to explain the lack of osteocytes. First, the absence of osteocytes in bone, even in a cellular-boned fish, is not unusual (Moss, 1961). In early life stages, when the bone is still very thin, cellular bone is often acellular (Huysseune, 2000). Thus, the absence of osteocytes may not be a character of sufficient weight to reject a bone-like character. Second, the outside of the pedicel is covered by mesenchymal cells that are to be considered as genuine osteoblasts. That these osteoblasts do not become entrapped in the pedicel either may well depend on their gene expression profile, compared to the osteoblasts lining the supporting bone and that become entrapped as osteocytes (Franz-Odendaal et al., 2006). Recent insights also point to the role of the highly dynamic nature of collagen assembly and maturation in the entrapment of osteocytes (Shiflett et al., 2019). According to this scenario, possible subtle differences in the organization of the collagen in the pedicel versus the supporting bone may trigger differences in entrapment of osteocytes.

The small cell prolongations, not unlike dentinal tubules, extending into the pedicel, represent another feature that questions the rather osteoblastic nature of the cells depositing the pedicel. However, not even this character can be unambiguously attributed to odontoblasts. Such cell prolongations, issuing from cells that remain at the surface of the matrix deposited, are reminiscent of the canaliculi of Williamson, described in the bone of extant holosteans (e.g., *Amia*, *Lepisosteus*; Sire and Meunier, 1994). Their nature has been heavily debated – whether of osteoblastic or odontoblastic origin – but arguments prevail to consider them to have an osteoblastic origin (Sire and Meunier, 1994).

What can these results teach us about osteoblast/odontoblast dichotomy and differentiation pathways? Ørvig (1951) described how, in the case of osteodentin (as in *Esox*), osteoblast-like cells that send short branching processes into the matrix modify directly into odontoblasts with a long peripherically directed odontoblast process. Thus, Ørvig (1951, 1967) considered that, in the teeth of some actinopterygian fishes, osteoblasts may turn into odontoblasts, but argued that, conversely, odontoblasts never transform into osteoblasts. He regarded odontoblasts as a special kind of osteoblasts. It is indeed remarkable how similar both cells are functionally (An, 2018). Even today, very often no distinction is made between both cell types and reference is simply made to “osteo/odontogenic differentiation” (e.g., Lin et al., 2019). Ariffin et al. (2017) nevertheless list a number of differences between odontoblasts and osteoblasts. However, this is a largely mammalian-centered view with limited relevance to the current study. Sire and Kawasaki (2012) argue that bone and dentin are the expression of two developmental pathways from a unique cell population with common origin. These different pathways are dictated by the environment in which the cells reside: distance from a signaling center in the overlying epithelium, and presence of a vascular (pulp) cavity. Likewise, Hall and Witten (2007, 2019) view bone and dentin as a continuum with typical bone and typical orthodentin at both sides of the spectrum. Our expression data support these views: cells covered by epithelium (odontoblasts) are *scpp5*-positive and Zns-5 negative, while the cells that lie beyond the cervical loop and deposit the pedicel are *scpp5*-negative and Zns-5 positive, like osteoblasts.

The *scpp5* gene is also expressed in ameloblasts. This may not be too remarkable. Indeed, P/Q-rich SCPPs (to which SCPP5 belongs) are primarily deposited by dental epithelial cells (ameloblasts) and are employed to form the tooth surface (Kawasaki and Weiss, 2008). This is different from acidic SCPPs, which are principally secreted from mesenchymally derived osteoblasts, osteocytes, and/or odontoblasts and which are used for bone and dentin. Thus, it is rather surprising that *scpp5* is expressed in odontoblasts. In fact, based on the lack of expression of *scpp5* in odontoblasts of the gar, *Lepisosteus oculatus*, Kawasaki et al. (2021) suggest that *scpp5* expression is a derived character in teleost odontoblasts. On the other hand, enameloid is built on a collagenous matrix, and both ameloblasts and odontoblasts express collagen type 1 (as demonstrated for Atlantic salmon, Huysseune et al., 2008). Kawasaki (2009) already considered the shared expression of *COL1*, *SPARC*, *SCPP5*, and *SCPP1* in odontoblasts and IDE cells as an indication of the use of common ECM proteins in dentin and enameloid. Likewise, Kawasaki and Weiss (2008) supported the idea that the teleost IDE cell has a gene expression profile intermediate between an odontoblast and a (tetrapod) ameloblast and that enameloid and dentin are closely related “in mode of mineralization, tissue origin and constitutive proteins”. The same line of thoughts can be followed for Zns-5. Whether its expression in the ameloblasts is transient or constitutional, and what its function is, needs however to be further explored.

In conclusion, the cells that, in zebrafish, deposit the dentinous bone of the pedicel have a molecular signature that approaches them to osteoblasts. Yet, the tissue they deposit does not truly qualify either as bone, or as dentin. Rather, the inference that the dentinous bone has characters that link it both to dentin (part of the tooth-forming developmental cascade, presence of cell prolongations), as well as to bone (lack of epithelial cover, expression profile of the scleroblasts, occasional presence of osteocytes) is in line with the conclusion of Kawasaki et al. (2009) that “there are only graded differences among bone, dentin, enameloid, and enamel, and these four tissues constitute an evolutionary continuum”. This view was also expressed by Kerebel et al. (1979), Hall and Witten (2007), and Sire and Kawasaki (2012), among others. Sire and Kawasaki (2012) furthermore suggested that osteoblasts and odontoblasts differentiated from a same cell population in the earliest vertebrates. Comparative developmental studies of dermal skeletal elements in extant species support this view (Sire and Huysseune, 2003). The latter authors considered the vicinity of the epithelium as a decisive factor in which developmental pathway is chosen. The dentinous bone discussed here may well be in support of this view, given that the expression profile of the scleroblasts changes at the very point where the epithelium is not present anymore. On the other hand, dentinous bone is just one of the possible tissue types that make up the pedicel in teleosts. Clearly, the evolutionary history of the attachment tissue as an entity requires further studies. Given that *scpp5* has also been found in the gar, *L. oculatus*, a basal actinopterygian (Kawasaki et al., 2017), as well as in highly evolved teleosts, such as fugu (*Takifugu rubripes*; Kawasaki and Weiss, 2006; where it is also expressed in IDE and odontoblasts), the use of this gene opens interesting perspectives for tracing the evolutionary history of attachment tissues in actinopterygians. Finally, it can also serve in studies of miniaturized teeth, such as found in the teleost medaka (*Oryzias latipes*), where a scleroblast of elusive identity complements the single odontoblast present (Larionova et al., 2021).

## Data Availablity Statement

The original contributions presented in the study are included in the article, further inquiries can be directed to the corresponding author.

## Ethics Statement

Ethical review and approval was not required for the animal study because the experimental protocols involved euthanasia only (no animal experiments); in such a case no approval of the ethical committee is required. All animal procedures used in this study were approved by Flemish authorities (laboratory permit number LA1400452).

## Author Contributions

AH designed the research. JTR carried out the research. JTR, PEW, and AH analyzed the results and corrected and finalized the manuscript. JTR and AH drafted the manuscript. All authors contributed to the article and approved the submitted version.

## Funding

JTR and AH acknowledge a grant of the Ghent University Research Fund (n° BOF24J2015001401).

## Conflict of Interest

The authors declare that the research was conducted in the absence of any commercial or financial relationships that could be construed as a potential conflict of interest.

## Publisher’s Note

All claims expressed in this article are solely those of the authors and do not necessarily represent those of their affiliated organizations, or those of the publisher, the editors and the reviewers. Any product that may be evaluated in this article, or claim that may be made by its manufacturer, is not guaranteed or endorsed by the publisher.

## Footnotes

^1^ www.zeiss.com
